# Albumin corrected anion gap for predicting in-hospital death among patients with acute myocardial infarction: A retrospective cohort study

**DOI:** 10.1016/j.clinsp.2024.100455

**Published:** 2024-07-29

**Authors:** Zhouzhou Lu, Yiren Yao, Yangyang Xu, Xin Zhang, Jing Wang

**Affiliations:** a Department of Cardiology, The Affiliated Huaian No.1 People's Hospital of Nanjing Medical University, Jiangsu Province, PR China; b The Second Clinical Medicine School, Nanjing Medical University, Nanjing, PR China

**Keywords:** Anion gap, Albumin corrected anion gap, Acute myocardial infarction, In-hospital death, Nomogram

## Abstract

**Objective:**

To explore the relationship between Anion Gap (AG), Albumin Corrected AG (ACAG), and in-hospital mortality of Acute Myocardial Infarction (AMI) patients and develop a prediction model for predicting the mortality in AMI patients.

**Methods:**

This was a retrospective cohort study based on the Medical Information Mart for Intensive Care (MIMIC)-Ⅲ, MIMIC-IV, and eICU Collaborative Study Database (eICU). A total of 9767 AMI patients who were admitted to the intensive care unit were included. The authors employed univariate and multivariable cox proportional hazards analyses to investigate the association between AG, ACAG, and in-hospital mortality; *p* < 0.05 was considered statistically significant. A nomogram incorporating ACAG and clinical indicators was developed and validated for predicting mortality among AMI patients.

**Results:**

Both ACAG and AG exhibited a significant association with an elevated risk of in-hospital mortality in AMI patients. The C-index of ACAG (C-index = 0.606) was significantly higher than AG (C-index = 0.589). A nomogram (ACAG combined model) was developed to predict the in-hospital mortality for AMI patients. The nomogram demonstrated a good predictive performance by Area Under the Curve (AUC) of 0.763 in the training set, 0.744 and 0.681 in the external validation cohort. The C-index of the nomogram was 0.759 in the training set, 0.756 and 0.762 in the validation cohorts. Additionally, the C-index of the nomogram was obviously higher than the ACAG and age shock index in three databases.

**Conclusion:**

ACAG was related to in-hospital mortality among AMI patients. The authors developed a nomogram incorporating ACAG and clinical indicators, demonstrating good performance for predicting in-hospital mortality of AMI patients.

## Highlights

• To explore the relationship between AG, ACAG and in-hospital mortality of AMI.
• Developing a prediction model for predicting the mortality in AMI patients.
• Demonstrating good predicting performance for in-hospital mortality of AMI.

## Introduction

Acute Myocardial Infarction (AMI) is characterized by myocardial necrosis resulting from acute coronary artery occlusion.^1^ There is evidence indicating that AMI has emerged as a global public health concern, with an Intensive Care Unit (ICU) mortality rate of 14.3 %.^2^ Therefore, early identification and timely intervention in high-risk patients are imperative to reduce the mortality associated with AMI.

Anion Gap (AG), which represents the differences between unmeasured anions and cations, is determined by subtracting the sum of serum chloride and bicarbonate concentrations from the sum of sodium concentrations.^3^^,^^4^ AG reflects acid-base balance and plays an important role in the differential diagnosis of both the etiology and type of metabolic acidosis.^5^ Current research has found that the level of AG was associated with the prognosis of many diseases, including cerebral infarction,^6^ coronary artery disease,^7^ sepsis,^8^ cardiogenic shock^9^ and AMI.^10^ These findings indicated that AG holds potential as a user-friendly clinical tool for disease prediction. Xu CB, et al., reported that higher AG was significantly related to an increased 30-day, 180-day, and 1-year all-cause mortality in AMI patients,^10^ However, previous studies have also highlighted that hypoalbuminemia generally leads to a decrease in the "normal" measured AG, thereby masking elevated AG levels.^11^, ^12^, ^13^, ^14^ Hatherill and colleagues proposed that Albumin Corrected Anion Gap (ACAG) represents a more appropriate clinical tool for diagnosing metabolic acidosis in the ICU.^15^ In the study of Hu TY, et al., ACAG was reported to have a better predictive value in comparison to AG for predicting in-hospital mortality among sepsis patients in the ICU.^16^ However, to our knowledge, few epidemiological studies have compared the prognostic value of AG and ACAG in relation to mortality among AMI patients.

Herein, the purpose of this study is as follows: (1) To explore the relationship between AG, ACAG, and in-hospital mortality in AMI patients, (2) To compare the predictive ability of AG and ACAG, and (3) To develop a prediction model for predicting the mortality among AMI patients.

## Methods

### Data sources

This retrospective cohort study was conducted utilizing three large and public critical care databases: the Medical Information Mart for Intensive Care (MIMIC)-Ⅲ, MIMIC-IV, and eICU Collaborative Study Database (eICU). This retrospective cohort study followed the STROBE Statement. MIMIC-Ⅲ, a single-center database, contains comprehensive and de-identified data associated with patients admitted to ICU at Beth Israel Deaconess Medical Center from 2001 to 2012.^17^ MIMIC-IV, in brief, is an updated version of MIMIC-III, containing data on patients admitted to the ICU from 2008 to 2019.^18^ eICU is a multi-center ICU database with high-granularity data, encompassing de-identified data related to more than 200,000 ICU admissions in the United States during the period spanning from 2014 to 2015.^19^ Since patients' information has been de-identified in all three databases and ethical approval has been obtained from both the Institutional Review Boards and the Massachusetts Institute of Technology, obtaining informed consent is not deemed necessary for this study.

### Study population

All patients who met the following criteria in the MIMIC-III, MIMIC-IV, and eICU databases were included in this retrospective cohort study. Inclusion criteria: (1) Patients diagnosed with AMI; (2) Patients aged ≥18 years. Those patients who had incomplete information on AG or albumin were excluded.

### Data collection

The following data were recorded: (1) Demographic data: gender, age (years) and race; (2) Vital signs: Systolic Blood Pressure (SBP, mmHg), Diastolic Blood Pressure (DBP, mmHg), temperature (°C), heart rate (times/min) and pulse oximetry-derived oxygen saturation (SPO_2_, %); (3) Laboratory parameters: AG, ACAG, potassium (mEq/L), magnesium (mg/dL), glucose (mg/dL), creatinine (mg/dL), Blood Urea Nitrogen (BUN, mg/dL), Red Blood cell distribution Width (RDW, %), White Blood Cell (WBC, K/µL), Red Blood Cells (RBC, m/µL) and hemoglobin (g/dL); (4) Medical history: Congestive Heart Failure (CHF), Atrial Fibrillation (AF), diabetes, valval disorder, Peripheral Vascular Disease (PVD), cardiogenic shock, malignant cancer, arrhythmias, AMI type; (5) Vasopressor use, thrombolysis, Percutaneous Coronary Intervention (PCI), antiplatelet drug, statins, anticoagulant, albumin (g/dL), AG (mEq/L), and age shock index. Noticeably, only patients’ data of the first ICU experience at the first admission were analyzed; this study was analyzed using vital signs and laboratory parameters recorded within 24 hours of initial admission. AG were calculated by using the formulae: AG(mmoL/L)=plasmasodium(mmoL/L)−[plasmachloride(mmoL/L)+plasmatotalbicarbonate(mmoL/L)];ACAGwerecalculated:ACAG(mmoL/L)=AG+2.5×(4−serumalbuminlevel(g/dL).^20^ The authors adopted the maximally selected method to determine the optimal cut-off value of AG, albumin, and ACAG.^21^ The ACAG was divided into high-level (≥ 19.24 mmoL/L) and low-level groups (< 19.24 mmoL/L). The survival curves were plotted using the Kaplan-Meier (KM) method in three databases and subsequently compared between the two groups using the log-rank test (Supplemental Fig. 1). The primary outcome of the present study was in-hospital mortality occurrence in patients with AMI.

### Development and validation of the prediction model

In the present study, the subjects from the MIMIC-III database were utilized as the cohort for establishing the prediction model, while those from the MIMIC-IV and eICU databases served as external validation cohorts, respectively. The authors adopted univariate and multivariable cox proportional hazards models to identify predictors associated with the mortality of AMI patients from the MIMIC-III database. The prediction model based on the predictors and ACAG (ACAG combined model) was conducted to assess the mortality risk among AMI patients. The prediction performance of the developed ACAG combined model was evaluated in the three databases by Concordance-index (C-index), and Area Under the Curve (AUC) of Receiver-Operating Characteristic (ROC) curves. A nomogram was developed to visualize the prediction model. In addition, the authors also compared the predictive value between the developed ACAG combined model and other models (ACAG and age shock index) for mortality among AMI patients.

### Statistical analysis

The normality of continuous variables was assessed using skewness and kurtosis, while the homogeneity of variance was tested using Levene's test. Continuous variables with normal distribution were described as Mean ± standard deviation (Mean ± SD). Group comparisons with both normal distribution and homogeneity of variance were conducted using ANOVA. Group comparisons with normal distribution but heterogeneity of variance were performed using the One-Way test. Continuous variables with non-normal distribution were expressed as median and interquartile range M (Q1, Q3). Kruskal-Wallis H rank sum test was used for comparison between groups. Categorical variables were described as a number of cases and constituent ratio n (%), and the Chi-Squared test was used for comparison. Supplemental Table 1 shows the post hoc test.

Univariable and multivariable cox proportional hazard models were used for exploring the relationship between AG, ACAG and in-hospital mortality among AMI patients, and Hazard Ratio (HR) with a 95% Confidence Interval (95% CI) was calculated. Model 1 was a coarse model with unadjusted variables; Model 2 adjusted gender; Model 3 adjusted gender, potassium, creatinine, BUN, RDW, WBC, AF, diabetes, cardiogenic shock, vasopressor use, AMI type, antiplatelet drug, statins, and age shock index. Then, the authors compared the prediction performance of a single indicator: AG and ACAG. Additionally, the authors developed and validated an ACAG combined model to predict the in-hospital mortality risk in AMI patients. Multiple imputation was performed using R MICE for missing values (Supplemental Table 2). Comparisons between groups and multivariable analyses were performed using SAS 9.4 (SAS Institute Inc., Cary, NC, USA), and all other analyses were performed using *R* (version 4.2.0). The survival_ROC package was employed for the development of the prediction model; p < 0.05 was considered statistically significant.

## Results

### Baseline characteristics

**Fig. 1:**
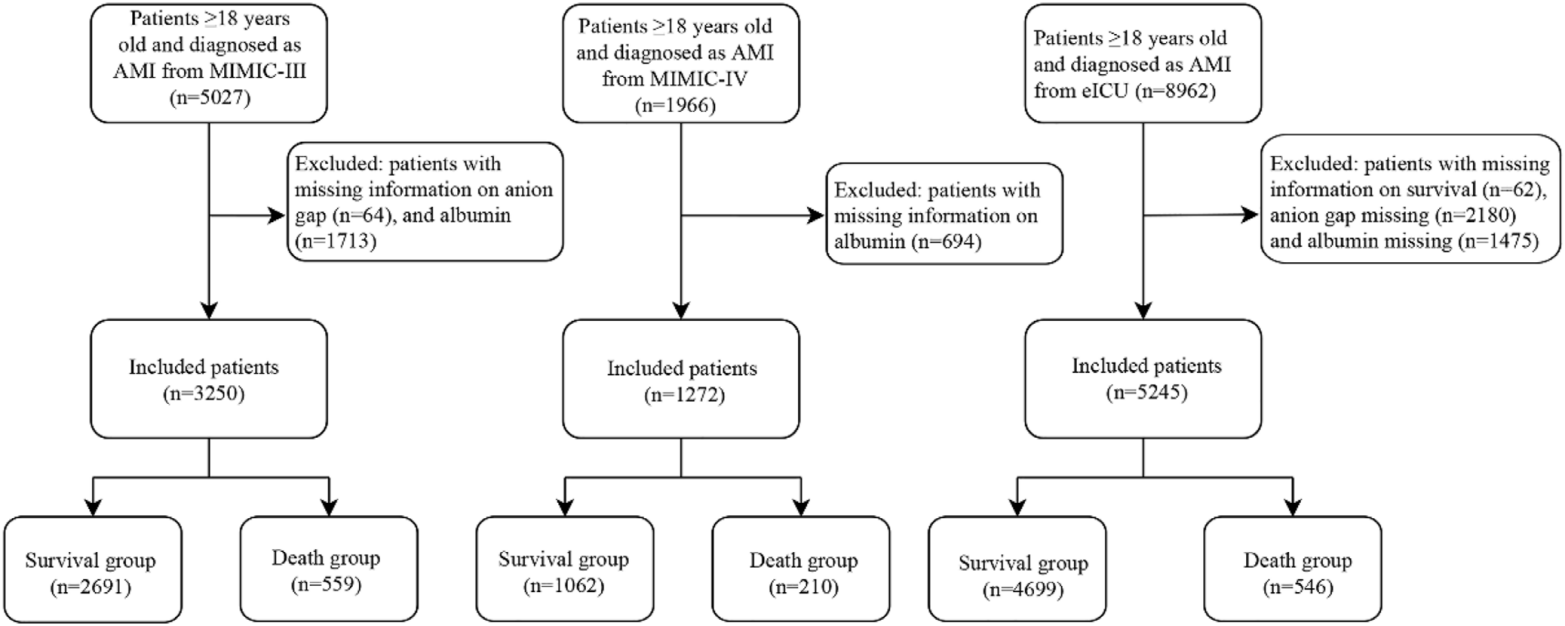
Flow chart for the selection of participants in the study.

After excluding some patients who were <18 years old and had incomplete information about AG or albumin, 3250 AMI patients from the MIMIC-III database were included as the training set in this study, 1272 AMI patients from the MIMIC-IV database, and 5245 AMI patients from the eICU database were included as the external validation cohorts (testing set) (Fig. 1). The end time of follow-up was discharge or death. The median follow-up was 7.58 (4.87, 13.17) days in the MIMIC-III database, 7.84 (5.11, 13.29) days in the MIMIC-IV database, and 3.95 (2.20, 7.81) days in the eICU database. Table 1 presents the baseline characteristics of the study population in each dataset, and the differences in characteristics in the three datasets were compared. Notably, most variables had statistical differences between the MIMIC-III, MIMIC-IV, and eICU databases (p < 0.05).

**Table 1:** Baseline characteristics in three datasets.

Variables	Total (*n* = 9767)	MIMIC-III (*n* = 3250)	MIMIC-IV (*n* = 1272)	Eicu (*n* = 5245)	p
Age, years, Mean (±SD)	68.61 (± 13.16)	70.97 (± 12.81)	70.24 (± 12.36)	66.76 (± 13.27)	<0.001
Gender, n (%)					0.008
Female	3708 (37.96)	1300 (40.00)	454 (35.69)	1954 (37.25)	
Male	6059 (62.04)	1950 (60.00)	818 (64.31)	3291 (62.75)	
Race, n (%)					<0.001
Asian	209 (2.14)	58 (1.78)	31 (2.44)	120 (2.29)	
Black/African American	702 (7.19)	210 (6.46)	78 (6.13)	414 (7.89)	
Other	893 (9.14)	163 (5.02)	116 (9.12)	614 (11.71)	
White	7963 (81.53)	2819 (86.74)	1047 (82.31)	4097 (78.11)	
CHF, yes, n (%)	2727 (27.92)	1755 (54.00)	222 (17.45)	750 (14.30)	<0.001
AF, yes, n (%)	755 (7.73)	157 (4.83)	105 (8.25)	493 (9.40)	<0.001
Diabetes, yes, n (%)	2290 (23.45)	977 (30.06)	541 (42.53)	772 (14.72)	<0.001
Valval disorder, yes, n (%)	1056 (10.81)	656 (20.18)	350 (27.52)	50 (0.95)	<0.001
PVD, yes, n (%)	550 (5.63)	428 (13.17)	85 (6.68)	37 (0.71)	<0.001
Cardiogenic shock, yes, n (%)	1096 (11.22)	486 (14.95)	225 (17.69)	385 (7.34)	<0.001
Malignant cancer, yes, n (%)	670 (6.86)	552 (16.98)	37 (2.91)	81 (1.54)	<0.001
Arrhythmias, yes, n (%)	3454 (35.36)	1583 (48.71)	836 (65.72)	1035 (19.73)	<0.001
Anemia, yes, n (%)	4946 (50.64)	1982 (60.98)	840 (66.04)	2124 (40.50)	<0.001
Heart rate, bpm, Mean (±SD)	85.37 (± 18.55)	87.35 (± 18.74)	85.96 (± 17.59)	84.00 (± 18.54)	<0.001
SBP, mmHg, Mean (±SD)	122.31 (±24.06)	122.11 (± 25.06)	119.68 (± 23.13)	123.07 (± 23.60)	<0.001
DBP, mmHg, Mean (±SD)	66.99 (± 16.81)	62.77 (± 16.85)	63.23 (± 16.79)	70.51 (± 15.97)	<0.001
SPO_2_, %, M (Q₁, Q₃)	98.00 (96.00, 100.00)	99.00 (96.00, 100.00)	99.00 (96.00, 100.00)	97.00 (95.40, 99.00)	<0.001
Temperature,°C, M (Q₁, Q₃)	36.60 (36.20, 36.90)	36.50 (35.90, 37.00)	36.56 (36.19, 36.96)	36.60 (36.40, 36.90)	<0.001
WBC, K/µL, M (Q₁, Q₃)	10.80 (8.10, 14.40)	10.80 (7.90, 14.60)	10.10 (7.50, 13.80)	10.90 (8.40, 14.40)	<0.001
RBC, K/µL, M (Q₁, Q₃)	10.00 (3.98, 20.00)	3.93 (3.47, 4.43)	3.87 (3.36, 4.39)	19.00 (14.00, 30.00)	<0.001
Creatinine, mg/dL, M (Q₁, Q₃)	1.10 (0.89, 1.61)	1.10 (0.90, 1.70)	1.10 (0.90, 1.70)	1.10 (0.87, 1.53)	<0.001
BUN, mg/dL, Mean (±SD)	28.29 (± 21.04)	31.80 (± 23.01)	30.22 (± 20.82)	25.65 (± 19.38)	<0.001
Glucose, mg/dL, M (Q₁, Q₃)	141.00 (113.00, 195.00)	142.00 (112.00, 197.75)	133.50 (107.00, 182.00)	141.00 (114.00, 196.00)	<0.001
Magnesium, mg/dL, M (Q₁, Q₃)	1.90 (1.72, 2.10)	1.90 (1.70, 2.20)	2.00 (1.80, 2.20)	1.90 (1.70, 2.10)	<0.001
Potassium, mEq/L, Mean (±SD)	4.22 (±0.74)	4.33 (±0.81)	4.28 (±0.70)	4.13 (±0.70)	<0.001
RDW, %, Mean (±SD)	14.56 (± 1.90)	14.59 (± 1.90)	14.69 (± 1.89)	14.50 (± 1.91)	0.003
AMI type, n (%)					<0.001
NSTEMI	5886 (60.26)	2147 (66.06)	952 (74.84)	2787 (53.14)	
STEMI	1953 (20.00)	860 (26.46)	250 (19.65)	843 (16.07)	
Unknown	1928 (19.74)	243 (7.48)	70 (5.50)	1615 (30.79)	
Vasopressor, yes, n (%)	2571 (26.32)	1205 (37.08)	496 (38.99)	870 (16.59)	<0.001
Thrombolysis, yes, n (%)	94 (0.96)	48 (1.48)	14 (1.10)	32 (0.61)	<0.001
PCI, yes, n (%)	2240 (22.93)	883 (27.17)	221 (17.37)	1136 (21.66)	<0.001
Antiplatelet drug, yes, n (%)	4533 (46.41)	2019 (62.12)	1210 (95.13)	1304 (24.86)	<0.001
Statins, yes, n (%)	3998 (40.93)	1694 (52.12)	1136 (89.31)	1168 (22.27)	<0.001
Anticoagulant, yes, n (%)	2777 (28.43)	668 (20.55)	825 (64.86)	1284 (24.48)	<0.001
Follow time, days, M (Q₁, Q₃)	5.78 (3.03, 10.26)	7.58 (4.87, 13.17)	7.84 (5.11, 13.29)	3.95 (2.20, 7.81)	<0.001
Age shock index, score, Mean (±SD)	50.04 (± 19.22)	53.03 (± 19.60)	52.12 (± 17.06)	47.67 (± 19.15)	<0.001
AG, mEq/L, Mean (±SD)	13.91 (± 5.06)	16.29 (± 4.50)	15.52 (± 4.15)	12.05 (± 4.83)	<0.001
AG, n (%)					<0.001
< 18	7864 (80.52)	2240 (68.92)	958 (75.31)	4666 (88.96)	
≥ 18	1903 (19.48)	1010 (31.08)	314 (24.69)	579 (11.04)	
Albumin, g/dL, Mean (±SD)	3.37 (± 0.65)	3.33 (± 0.62)	3.39 (± 0.64)	3.40 (± 0.67)	<0.001
Albumin, n (%)					0.338
< 3	2371 (24.28)	818 (25.17)	300 (23.58)	1253 (23.89)	
≥ 3	7396 (75.72)	2432 (74.83)	972 (76.42)	3992 (76.11)	
ACAG, Mean (±SD)	15.42 (± 5.33)	17.90 (± 4.75)	16.99 (± 4.58)	13.50 (± 5.07)	<0.001
ACAG, n (%)					<0.001
< 19.24	7829 (80.16)	2233 (68.71)	955 (75.08)	4641 (88.48)	
≥ 19.24	1938 (19.84)	1017 (31.29)	317 (24.92)	604 (11.52)	

CHF, Congestive Heart Failure; AF, Atrial Fibrillation; PVD, Peripheral Vascular Disease; SBP, Systolic Blood Pressure; DBP, Diastolic Blood Pressure; SPO_2_, Pulse Oximetry-derived Oxygen Saturation; WBC, White Blood Cell; RBC, Red Blood Cells; BUN, Blood Urea Nitrogen; RDW, Red Blood Cell Distribution width; AMI, Acute Myocardial Infarction; PCI, Percutaneous Coronary Intervention; AG, Anion Gap; ACAG, Albumin Corrected Anion Gap.

### The relationship between AG, ACAG and in-hospital mortality of AMI patients

The authors assessed the relationship between AG, ACAG and in-hospital mortality of AMI patients from the MIMIC-III database. Importantly, the relationship between AG, ACAG, and the in-hospital mortality among AMI patients is presented in Table 2. After adjusting all covariates (Model 3), the authors found that AMI patients with an ACAG ≥ 19.24 mmoL/L exhibited a 24% elevated risk of in-hospital mortality compared to those with an ACAG < 19.24 mmoL/L, and AG was also a risk factor for in-hospital mortality among AMI patients. The authors also compared the predicting performance of AG and ACAG for predicting in-hospital mortality for AMI patients. As shown in Table 3, the C-index of ACAG is 0.606 (95% CI 0.581‒0.630), which was significantly higher than AG (C-index = 0.589, 95%CI 0.565‒0.614). The result also indicated that ACAG might be a more appropriate predictor of in-hospital mortality among AMI patients compared to AG.

**Table 2:** The relationship between AG, ACAG and the in-hospital mortality of AMI patients.

	Model 1		Model 2		Model 3	
Variables	HR (95 % CI)	p	HR (95 % CI)	p	HR (95 % CI)	p
ACAG						
< 19.24	Ref		Ref		Ref	
≥ 19.24	1.86 (1.57‒2.20)	< 0.001	1.84 (1.56‒2.18)	< 0.001	1.24 (1.02‒1.50)	0.028
AG						
< 18	Ref		Ref		Ref	
≥ 18	1.71 (1.45‒2.02)	< 0.001	1.70 (1.44‒2.01)	< 0.001	1.21 (1.00‒1.47)	0.046

AMI, Acute Myocardial Infarction; AG, Anion Gap; ACAG, Albumin Corrected Anion Gap; RR, Relative Risk; CI, Confidence Interval; Model 1, A coarse model with unadjusted variables; Model 2, Adjusted gender; Model 3, Adjusted gender, potassium, creatinine, blood urea nitrogen, red blood cell distribution width, white blood cell, atrial fibrillation, diabetes, cardiogenic shock, vasopressor use, AMI type, antiplatelet drug, statins, and age shock index.

**Table 3:** The prediction value of single indicator

	MIMIC-Ⅲ
Indicator	C-index (95 % CI)
AG	0.589 (0.565‒0.614)
ACAG	0.606 (0.581‒0.63)
Statistic	2.19
p	0.028

AG., Anion Gap; ACAG, Albumin Corrected Anion Gap.

### Development of nomogram (ACAG combined model)

**Fig. 2:**
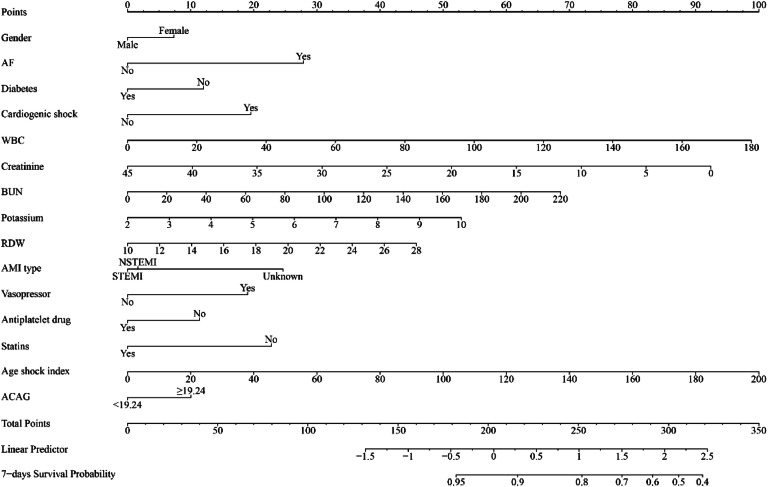
The nomogram for prediction of in-hospital mortality among AMI patients. AMI, Acute Myocardial Infarction; AF, Atrial Fibrillation; RDW, Red blood cell Distribution Width; BUN, Blood Urea Nitrogen; WBC, White Blood Cell; ACAG, Albumin Corrected Anion Gap.

Some important clinical indicators were screened by univariate and multivariate cox proportional hazards analyses and included in the prediction model (Supplemental Table 3). A nomogram incorporating ACAG and several clinical indicators was developed to predict the risk of in-hospital mortality among AMI patients. Figure 2 illustrates the fifteen indicators included in the nomogram: ACAG, gender, potassium, creatinine, BUN, RDW, WBC, AF, diabetes, cardiogenic shock, vasopressor use, AMI type, antiplatelet drug, statins, and age shock index.

### Validation of nomogram

**Fig. 3:**
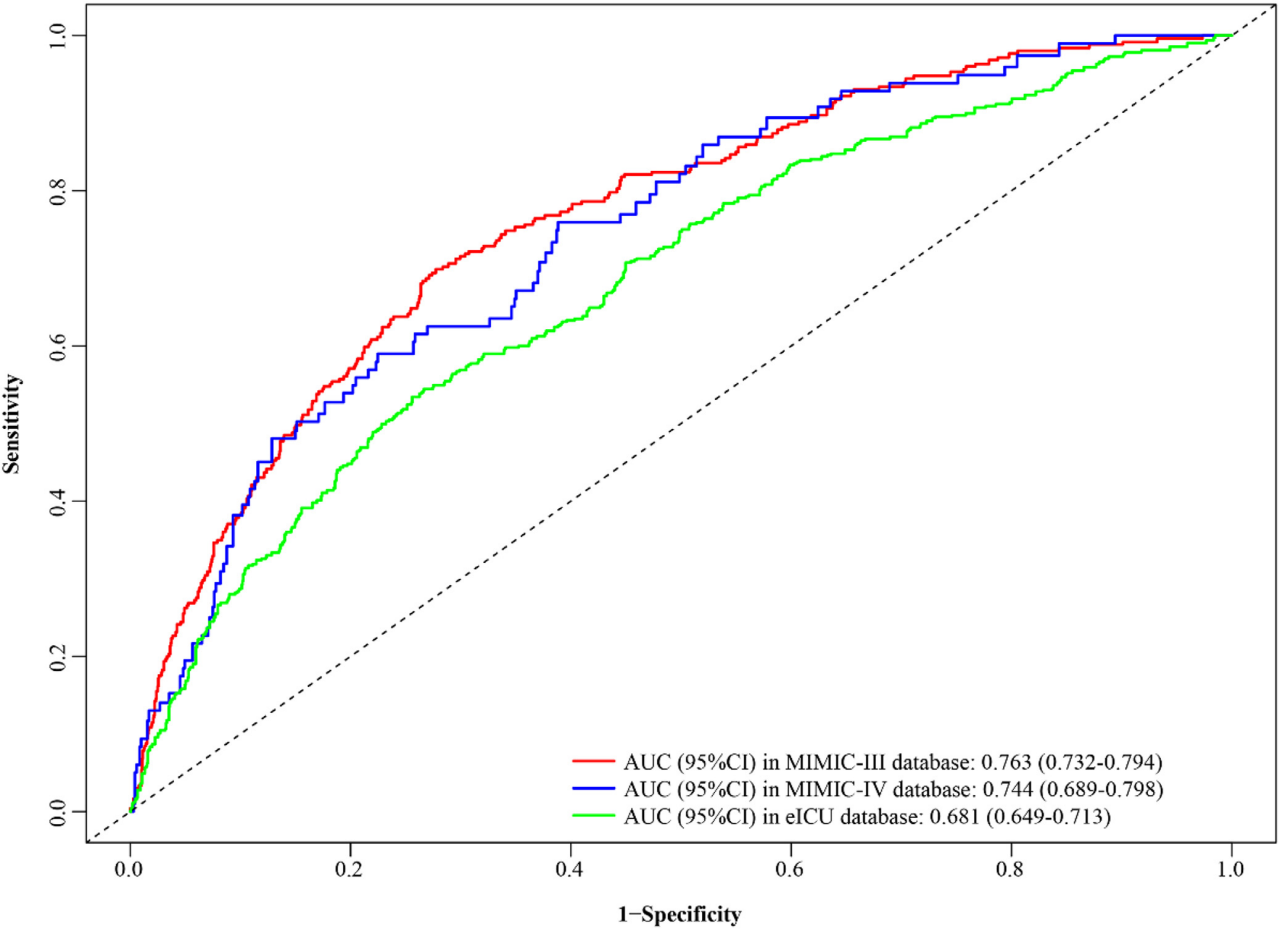
Receiver operating characteristic curves of the nomogram in the training set and validation cohorts.

The C-index of the nomogram was 0.759 (95% CI 0.738‒0.781) in the training set (MIMIC-III), 0.756 (95% CI 0.720‒0.792) (MIMIC-IV), and 0.762 (95% CI 0.740‒0.783) (eICU) in the validation cohorts (Table 4), which indicated that the nomogram had a favorable prediction ability. As shown in Figure 3, AUC of the nomogram was 0.763 (95% CI 0.732‒0.794) in the MIMIC-III cohort, 0.744 (95% CI 0.689‒0.798) in the MIMIC-IV cohort and 0.681 (95% CI 0.649‒0.713) in the eICU cohort. In addition, the authors also compared the predicting performance of the nomogram (ACAG combined model) and other models (ACAG and age shock index) in predicting the risk of in-hospital mortality for AMI patients (Table 5). The C-index of the nomogram was 0.759 (95% CI 0.738‒0.781), which is obviously higher than ACAG 0.606 (95% CI 0.581‒0.630), and age shock index 0.628 (95% CI 0.600‒0.656) in the MIMIC-III database. Simultaneously, the authors found that the C-index of the nomogram was 0.756 (95% CI 0.720‒0.792), which was also obviously higher than ACAG 0.610 (95% CI 0.569‒0.651), and age shock index 0.589 (95% CI 0.541‒0.636) in the MIMIC- IV database. Similar results were found in the eICU database.

**Table 4:** Prediction performance of nomogram.

	C-index (95 % CI)
**MIMIC-III**	0.759 (0.738‒0.781)
**MIMIC-IV**	0.756 (0.72‒0.792)
**eICU**	0.762 (0.74‒0.783)

CI, Confidence Interval.

**Table 5:** Comparison of models in predicting the in-hospital mortality of patients with AMI.

	MIMIC-III	MIMIC-IV	eICU
Prediction models	C-index (95 % CI)	C-index (95 % CI)	C-index (95 % CI)
ACAG	0.606 (0.581‒0.63)^a^	0.61 (0.569‒0.651)^a^	0.605 (0.582‒0.627)^a^
Nomogram	0.759 (0.738‒0.781)	0.756 (0.72‒0.792)	0.762 (0.74‒0.783)
Age shock index	0.628 (0.6‒0.656)^a^	0.589 (0.541‒0.636)^a^	0.704 (0.68‒0.729)^a^

CI, Confidence Interval.

a Indicates statistically significant differences between the nomogram and other models, with a *p* < 0.001.

## Discussion

In the current study, the authors found that both ACAG and AG were significantly associated with an increased risk of in-hospital mortality among AMI patients. Notably, ACAG exhibited a potentially higher predictive value than AG for the prediction of in-hospital mortality. In addition, the authors developed a predicting nomogram (ACAG combined model) incorporating ACAG, gender, potassium, creatinine, BUN, RDW, WBC, AF, diabetes, cardiogenic shock, vasopressor use, AMI type, antiplatelet drug, statins, and age shock index. The developed nomogram (ACAG combined model) had a good predictive ability for in-hospital mortality among AMI patients. To further verify the predictive performance of the nomogram, the authors conducted external validation using datasets from two additional public databases. These findings suggested that the nomogram based on ACAG and clinical indicators may be a good tool.

In recent years, there has been extensive investigation into the association between ACAG levels and various diseases. Hu TY et al. conducted a retrospective propensity score matching analysis and concluded that ACAG was related to in-hospital mortality among intensive care patients with sepsis, and ACAG exhibits superior predictive value for in-hospital mortality of intensive care patients with sepsis compared to albumin and AG.^16^ Hagiwara S et al. investigated the correlation between ACAG and Return of Spontaneous Circulation (ROSC) in Patients with Cardiopulmonary Arrest (CPA), and pointed out that both AG and ACAG were related to ROSC, however, ACAG demonstrated superior predictive capability for ROSC in CPA patients compared to AG.^22^ These studies also indicated that ACAG may be a better predictor than AG. However, to the best of our knowledge, there was limited data on the ACAG in the prognosis of AMI patients so far. In this retrospective cohort study, the result showed that both ACAG and AG were risk factors for in-hospital mortality in AMI patients. In brief, the ACAG parameter, which is composed of albumin and AG, comprehensively reflects the levels of these two factors. Albumin and AG have been considered as the biomarkers of prognosis in AMI patients.^10^^,^^23^ The association of ACAG and in-hospital death in AMI might be explained by the inflammation.^10^ It is noteworthy that ACAG showed a higher predictive power compared to AG in this study, suggesting the potential of ACAG as a prognostic indicator for in-hospital mortality among AMI patients.

Previous studies have reported that the predictive value of a single biomarker is not good for clinical practice.^24^^,^^25^ Nomogram, as an easy-to-use prediction model, has been widely used to predict the prognosis of diseases.^26^, ^27^, ^28^, ^29^ In the present study, the authors developed a nomogram by combining ACAG and different clinical indexes to achieve a good predictive performance in predicting the probability of in-hospital mortality for AMI patients. This nomogram demonstrated excellent predictive accuracy for in-hospital mortality of AMI patients who were from MIMIC-III, MIMIC-IV, and eICU databases.

To our knowledge, this is the first study on assessing the relationship between ACAG and in-hospital mortality for AMI patients and developing a nomogram by combining ACAG with different clinical indexes. ACAG is an easy-to-measure index that indicates the applicability of the developed nomogram. Also, the authors also performed an external validation using two large public databases to assess the predictive ability of the nomogram. The nomogram based on ACAG and clinical indicators could serve as a valuable tool in identifying AMI patients at high risk of in-hospital mortality and aiding clinicians in customizing precise management strategies and therapies for them. However, the authors must acknowledge some limitations of this study. Firstly, the authors excluded several AMI patients with incomplete information, which may affect the present results. Secondly, some clinical indicators that may be related to AMI were not included in the analysis due to excessive missing in the database, such as Creatine Kinase-MB (CK-MB) and troponin levels.^30^^,^^31^ In addition, these databases (MIMIC-III, MIMIC-IV and eICU) Lacked left Ventricular Ejection Fraction (LVEF) Killip class at presentation, severity of coronary disease of patients. Lastly, this study collected the data of the patients from MIMIC-III, MIMIC-IV and eICU databases, and only AMI patients in ICU were considered. The authors cannot confirm whether this nomogram is applicable to AMI patients who were not admitted to the ICU. The results should be interpreted with caution. More prospective clinical trials are needed to verify this finding in the future and explore the mechanism underlying the prognostic relationship between ACAG and AMI patients.

## Conclusion

In short, higher ACAG level was associated with increased in-hospital mortality in AMI patients. ACAG may possess a higher predictive value than AG in predicting in-hospital mortality among AMI patients. Moreover, a nomogram integrating ACAG and clinical parameters (gender, potassium, creatinine, BUN, RDW, WBC, AF, diabetes, cardiogenic shock, vasopressor use, AMI type, antiplatelet drug, statins, and age shock index) was developed and external validated. This nomogram shows a higher predicting performance than ACAG and age shock index.

## Declarations

Ethics approval and consent to participate: Not applicable, because three databases belong to public databases, the patients involved in the database have obtained ethical approval from the Institutional Review Boards and Massachusetts Institute of Technology, and users can download relevant data for free for research and publish relevant articles, and the present study is based on open-source data, and the Affiliated Huaian No.1 People's Hospital of Nanjing Medical University & The Second Clinical Medicine School, Nanjing Medical University, do not require research using publicly available data to be submitted for review to their ethics committee, so there are no ethical issues and other conflicts of interest.

## Consent for publication

Not applicable.

## Availability of data and materials

The datasets used and/or analyzed during the current study are available from the corresponding author upon reasonable request.

## Funding

None.

## CRediT authorship contribution statement

**Zhouzhou Lu:** Conceptualization, Data curation, Formal analysis, Writing – original draft, Writing – review & editing. **Yiren Yao:** Conceptualization, Data curation, Formal analysis, Writing – original draft, Writing – review & editing. **Yangyang Xu:** Data curation, Formal analysis, Writing – review & editing. **Xin Zhang:** Data curation, Formal analysis, Writing – review & editing. **Jing Wang:** Conceptualization, Writing – review & editing.

## Declaration of competing interest

The authors declare no conflicts of interest.

## Appendix Supplementary materials

Image, application 1

## References

[1] Lindahl B, Mills NL. (2023). A new clinical classification of acute myocardial infarction. Nat Med.

[2] Valley TS, Iwashyna TJ, Cooke CR, Sinha SS, Ryan AM, Yeh RW (2019). Intensive care use and mortality among patients with ST elevation myocardial infarction: retrospective cohort study. BMJ.

[3] Chen J, Dai C, Yang Y, Wang Y, Zeng R, Li B (2022). The association between anion gap and in-hospital mortality of post-cardiac arrest patients: a retrospective study. Sci Rep.

[4] Sun X, Lu J, Weng W, Yan Q. (2023). Association between anion gap and all-cause mortality of critically ill surgical patients: a retrospective cohort study. BMC Surg.

[5] Wang H, Liu C, Xu H, Zhang Y, Gao P, Geng S (2022). The association between serum anion gap and all-cause mortality in cerebral infarction patients after treatment with RTPA: a retrospective analysis. Dis Markers.

[6] Liu X, Feng Y, Zhu X, Shi Y, Lin M, Song X (2020). Serum anion gap at admission predicts all-cause mortality in critically ill patients with cerebral infarction: evidence from the MIMIC-III database. Biomarkers.

[7] Wang XM, Deng YS, He B, Liu JW, Zhang ZH, Ye ZD (2023). The serum anion gap is associated with the prognosis of coronary artery bypass grafting (CABG): analysis based on the MIMIC-IV database. Eur Rev Med Pharmacol Sci.

[8] Mitra B, Roman C, Charters KE, O'Reilly G, Gantner D, Cameron PA. (2020). Lactate, bicarbonate and anion gap for evaluation of patients presenting with sepsis to the emergency department: A prospective cohort study. Emerg Med Australas.

[9] Zhang T, Wang J, Li X (2021). Association between anion gap and mortality in critically Ill patients with cardiogenic shock. Int J Gen Med.

[10] Xu C, Sun L, Dong M, Ullah H, Ullah H, Zhou J (2022). Serum anion gap is associated with risk of all-cause mortality in critically Ill patients with acute myocardial infarction. Int J Gen Med.

[11] Carvounis CP, Feinfeld DA. (2000). A simple estimate of the effect of the serum albumin level on the anion Gap. Am J Nephrol.

[12] Chawla LS, Shih S, Davison D, Junker C, Seneff MG. (2008). Anion gap, anion gap corrected for albumin, base deficit and unmeasured anions in critically ill patients: implications on the assessment of metabolic acidosis and the diagnosis of hyperlactatemia. BMC Emerg Med.

[13] Figge J, Jabor A, Kazda A, Fencl V. (1998). Anion gap and hypoalbuminemia. Crit Care Med.

[14] Zhao B, Li Y, Lang X, Fang S, Li Z, Li L (2023). Increased serum albumin corrected anion gap levels are associated with increased incidence of new-onset HF and poor prognosis in patients with acute myocardial infarction. Clin Chim Acta.

[15] Hatherill M, Waggie Z, Purves L, Reynolds L, Argent A (2002). Correction of the anion gap for albumin in order to detect occult tissue anions in shock. Arch Dis Child.

[16] Hu T, Zhang Z, Jiang Y. (2021). Albumin corrected anion gap for predicting in-hospital mortality among intensive care patients with sepsis: A retrospective propensity score matching analysis. Clin Chim Acta.

[17] Johnson AE, Pollard TJ, Shen L, Lehman LW, Feng M, Ghassemi M (2016). MIMIC-III, a freely accessible critical care database. Sci Data.

[18] Johnson AEW, Bulgarelli L, Shen L, Gayles A, Shammout A, Horng S (2023). MIMIC-IV, a freely accessible electronic health record dataset. Sci Data.

[19] Pollard TJ, Johnson AEW, Raffa JD, Celi LA, Mark RG, Badawi O. (2018). The EICU collaborative research database, a freely available multi-center database for critical care research. Sci Data.

[20] Aydın S, Aksakal E (2023). Relationship between albumin-corrected anion gap and mortality in hospitalized heart failure patients. Cureus.

[21] Laska E, Meisner M, Wanderling J. (2012). A maximally selected test of symmetry about zero. Statistics in medicine.

[22] Hagiwara S, Oshima K, Furukawa K, Nakamura T, Ohyama Y, Tamura J. (2013). The significance of albumin corrected anion gap in patients with cardiopulmonary arrest. Ann Thorac Cardiovasc Surg.

[23] Xia M, Zhang C, Gu J, Chen J, Wang LC, Lu Y (2018). Impact of serum albumin levels on long-term all-cause, cardiovascular, and cardiac mortality in patients with first-onset acute myocardial infarction. Clin Chim Acta.

[24] Vincent JL, Bogossian E, Menozzi M. (2020). The future of biomarkers. Crit Care Clin.

[25] Ding C, Hu T. (2021). Development and external verification of a nomogram for patients with persistent acute kidney injury in the intensive care unit. Int J Gen Med.

[26] Wu J, Zhang H, Li L, Hu M, Chen L, Xu B (2020). A nomogram for predicting overall survival in patients with low-grade endometrial stromal sarcoma: A population-based analysis. Cancer Commun (Lond).

[27] Lv J, Liu YY, Jia YT, He JL, Dai GY, Guo P (2021). A nomogram model for predicting prognosis of obstructive colorectal cancer. World J Surg Oncol.

[28] Cheng H, Xu JH, Kang XH, Liu XM, Wang HF, Wang ZX (2023). Nomogram for predicting the preoperative lymph node metastasis in resectable pancreatic cancer. J Cancer Res Clin Oncol.

[29] Chen Y, Xie K, Han Y, Xu Q, Zhao X. (2023). An easy-to-use nomogram based on SII and SIRI to predict in-hospital mortality risk in elderly patients with acute myocardial infarction. J Inflamm Res.

[30] Chen Y, Zhou X, Chen Z, Xia J, Guan F, Li Y (2023). The use of high-sensitivity cardiac troponin T and creatinine kinase-MB as a prognostic markers in patients with acute myocardial infarction and chronic kidney disease. Ren Fail.

[31] Liu Y, Tang XL, Ni Y, Duan LZ, Jing FJ. (2024). Diagnostic value of the creatine kinase-MB/creatine kinase and neutrophil/lymphocyte ratios in acute myocardial infarction. J Cardiothorac Surg.

