# Supplementary material for: Albumin corrected anion gap for predicting in-hospital death among patients with acute myocardial infarction: A retrospective cohort study
Source: Clinics (Sao Paulo). 2024 Jul 29;79:100455. doi: 10.1016/j.clinsp.2024.100455 (PMC11334651; doi:10.1016/j.clinsp.2024.100455)
Supplement: Supplementary file 1 [file mmc1.docx]

**CLINICS-D-24-00269_Supplementary Material**

**Supplemental Table 1** Post hoc test.

|  | **eICU** | **MIMIC-Ⅲ** | **Variables** | **Method** |
| --- | --- | --- | --- | --- |
| MIMIC-Ⅲ | 0.090 |  | RDW | ANOVA |
| MIMIC-IV | 0.005 | 0.381 | RDW | ANOVA |
| MIMIC-Ⅲ | <0.001 |  | Age | One-Way test |
| MIMIC-IV | <0.001 | 0.269 | Age | One-Way test |
| MIMIC-Ⅲ | <0.001 |  | Heart rate | One-Way test |
| MIMIC-IV | 0.002 | 0.069 | Heart rate | One-Way test |
| MIMIC-Ⅲ | 0.218 |  | SBP | One-Way test |
| MIMIC-IV | <0.001 | 0.007 | SBP | One-Way test |
| MIMIC-Ⅲ | <0.001 |  | DBP | One-Way test |
| MIMIC-IV | <0.001 | 1.000 | DBP | One-Way test |
| MIMIC-Ⅲ | <0.001 |  | BUN | One-Way test |
| MIMIC-IV | <0.001 | 0.065 | BUN | One-Way test |
| MIMIC-Ⅲ | <0.001 |  | Potassium | One-Way test |
| MIMIC-IV | <0.001 | 0.127 | Potassium | One-Way test |
| MIMIC-Ⅲ | <0.001 |  | Albumin | One-Way test |
| MIMIC-IV | 1.000 | 0.012 | Albumin | One-Way test |
| MIMIC-Ⅲ | <0.001 |  | AG | One-Way test |
| MIMIC-IV | <0.001 | <0.001 | AG | One-Way test |
| MIMIC-Ⅲ | <0.001 |  | ACAG | One-Way test |
| MIMIC-IV | <0.001 | <0.001 | ACAG | One-Way test |
| MIMIC-Ⅲ | <0.001 |  | Age shock index | One-Way test |
| MIMIC-IV | <0.001 | 0.450 | Age shock index | One-Way test |
| MIMIC-Ⅲ | 0.001 |  | SPO_2_ | Kruskal-Wallis H rank sum test |
| MIMIC-IV | <0.001 | 0.376 | SPO_2_ | Kruskal-Wallis H rank sum test |
| MIMIC-Ⅲ | <0.001 |  | Temperature | Kruskal-Wallis H rank sum test |
| MIMIC-IV | 1.000 | 0.001 | Temperature | Kruskal-Wallis H rank sum test |
| MIMIC-Ⅲ | 1.000 |  | WBC | Kruskal-Wallis H rank sum test |
| MIMIC-IV | 0.191 | 0.383 | WBC | Kruskal-Wallis H rank sum test |
| MIMIC-Ⅲ | <0.001 |  | RBC | Kruskal-Wallis H rank sum test |
| MIMIC-IV | <0.001 | 1.000 | RBC | Kruskal-Wallis H rank sum test |
| MIMIC-Ⅲ | <0.001 |  | Creatinine | Kruskal-Wallis H rank sum test |
| MIMIC-IV | 0.048 | 1.000 | Creatinine | Kruskal-Wallis H rank sum test |
| MIMIC-Ⅲ | 0.017 |  | Magnesium | Kruskal-Wallis H rank sum test |
| MIMIC-IV | <0.001 | 0.004 | Magnesium | Kruskal-Wallis H rank sum test |
| MIMIC-Ⅲ | 1.000 |  | Glucose | Kruskal-Wallis H rank sum test |
| MIMIC-IV | <0.001 | 0.001 | Glucose | Kruskal-Wallis H rank sum test |
| MIMIC-Ⅲ | <0.001 |  | Follow time | Kruskal-Wallis H rank sum test |
| MIMIC-IV | <0.001 | 0.050 | Follow time | Kruskal-Wallis H rank sum test |

SBP, Systolic Blood Pressure; DBP, Diastolic Blood Pressure; SPO_2_, Pulse oximetry-derived Oxygen Saturation; BUN, Blood Urea Nitrogen; RDW, Red blood cell Distribution Width; WBC, White Blood Cell; RBC, Red Blood Cells; AG, Anion Gap; ACAG, Albumin Corrected Anion Gap.

**Supplemental Table 2** The number and percentage of missing variables.

| **Missing variables** | **Number** | **Percentage** |
| --- | --- | --- |
| ***MIMIC-III*** |  |  |
| SPO_2_ | 17 | 0.52% |
| Temperature | 17 | 0.52% |
| Heart rate | 14 | 0.43% |
| Respiratory rate | 14 | 0.43% |
| Magnesium | 5 | 0.15% |
| SBP | 14 | 0.43% |
| DBP | 14 | 0.43% |
| ***MIMIC-IV*** |  |  |
| Temperature | 5 | 0.39% |
| Ethnicity | 218 | 17.14% |
| ***eICU*** |  |  |
| SPO_2_ | 1163 | 22.17% |
| Temperature | 276 | 5.26% |
| Heart rate | 613 | 11.69% |
| RDW | 370 | 7.05% |
| Glucose | 31 | 0.59% |
| Ethnicity | 76 | 1.45% |
| SBP | 754 | 14.38% |
| DBP | 754 | 14.38% |
| Magnesium | 1024 | 19.52% |

SBP, Systolic Blood Pressure; DBP, Diastolic Blood Pressure; SPO_2_, Pulse oximetry-derived Oxygen Saturation; RDW, Red blood cell Distribution Width.

**Supplemental Table 3** Screening of predictors by univariable and multivariable cox proportional hazards models.

| **Variables** | **Model 1** | | **Model 2** | |
| --- | --- | --- | --- | --- |
|  | **HR (95% CI)** | **p** | **HR (95% CI)** | **p** |
| Age | 1.04 (1.03‒1.04) | <0.001 |  |  |
| Gender |  |  |  |  |
| Female | Ref |  | Ref |  |
| Male | 0.81 (0.68‒0.95) | 0.011 | 0.84 (0.71‒1.00) | 0.050 |
| Race |  |  |  |  |
| Asian | Ref |  |  |  |
| Black/African American | 1.08 (0.52‒2.22) | 0.840 |  |  |
| Other | 1.14 (0.54‒2.40) | 0.734 |  |  |
| White | 1.16 (0.60‒2.24) | 0.659 |  |  |
| CHF, yes | 0.86 (0.73‒1.02) | 0.079 |  |  |
| AF, yes | 1.71 (1.25‒2.33) | 0.001 | 1.81 (1.31‒2.50) | <0.001 |
| Diabetes, yes | 0.77 (0.63‒0.93) | 0.006 | 0.77 (0.63‒0.94) | 0.009 |
| Valval disorder, yes | 0.87 (0.70‒1.07) | 0.186 |  |  |
| PVD, yes | 0.82 (0.64‒1.05) | 0.119 |  |  |
| Cardiogenic shock, yes | 1.94 (1.62‒2.33) | <0.001 | 1.53 (1.25‒1.87) | <0.001 |
| Malignant cancer, yes | 1.18 (0.95‒1.45) | 0.134 |  |  |
| Arrhythmias, yes | 1.17 (0.99‒1.38) | 0.073 |  |  |
| Anemia, yes | 1.28 (1.07‒1.54) | 0.008 |  |  |
| Heart rate | 1.01 (1.00‒1.01) | <0.001 |  |  |
| SBP | 0.99 (0.99‒1.00) | 0.002 |  |  |
| DBP | 1.00 (0.99‒1.00) | 0.141 |  |  |
| SPO_2_ | 0.99 (0.98‒1.00) | 0.008 |  |  |
| Temperature | 0.98 (0.96‒1.00) | 0.103 |  |  |
| WBC | 1.01 (1.01‒1.02) | <0.001 | 1.01 (1.01‒1.02) | <0.001 |
| RBC | 0.87 (0.77‒0.97) | 0.014 |  |  |
| Creatinine | 1.03 (1.00‒1.06) | 0.023 | 0.96 (0.92‒1.01) | 0.145 |
| BUN | 1.01 (1.01‒1.01) | <0.001 | 1.01 (1.00‒1.01) | <0.001 |
| Magnesium | 1.15 (0.99‒1.34) | 0.073 |  |  |
| Glucose | 1.00 (1.00‒1.00) | 0.078 |  |  |
| Potassium | 1.22 (1.12‒1.33) | <0.001 | 1.17 (1.07‒1.28) | 0.001 |
| RDW | 1.09 (1.05‒1.13) | <0.001 | 1.05 (1.01‒1.10) | 0.010 |
| AMI type |  |  |  |  |
| NSTEMI | Ref |  | Ref |  |
| STEMI | 1.09 (0.89‒1.34) | 0.386 | 0.98 (0.79‒1.21) | 0.819 |
| Unknown | 2.09 (1.64‒2.66) | <0.001 | 1.64 (1.28‒2.10) | <0.001 |
| Vasopressor, yes | 1.94 (1.63‒2.31) | <0.001 | 1.52 (1.26‒1.84) | <0.001 |
| Thrombolysis, yes | 0.57 (0.26‒1.28) | 0.175 |  |  |
| PCI, yes | 0.83 (0.68‒1.03) | 0.087 |  |  |
| Antiplatelet drug, yes | 0.61 (0.51‒0.72) | <0.001 | 0.79 (0.64‒0.96) | 0.017 |
| Statins, yes | 0.49 (0.41‒0.58) | <0.001 | 0.61 (0.49‒0.75) | <0.001 |
| Anticoagulant, yes | 0.93 (0.76‒1.15) | 0.516 |  |  |
| Age shock index | 1.02 (1.01‒1.02) | <0.001 | 1.01 (1.01‒1.01) | <0.001 |

CHF, Congestive Heart Failure; AF, Atrial Fibrillation; PVD, Peripheral Vascular Disease; SBP, Systolic Blood Pressure; DBP, Diastolic Blood Pressure; SPO_2_, Pulse oximetry-derived Oxygen Saturation; WBC, White Blood Cell; RBC, Red Blood Cells; BUN, Blood Urea Nitrogen; RDW, Red Blood cell distribution Width; AMI, Acute Myocardial Infarction; PCI, Percutaneous Coronary Intervention.

**Supplemental Figure 1** Kaplan-Meier survival analysis curves for in-hospital mortality among AMI patients.

**
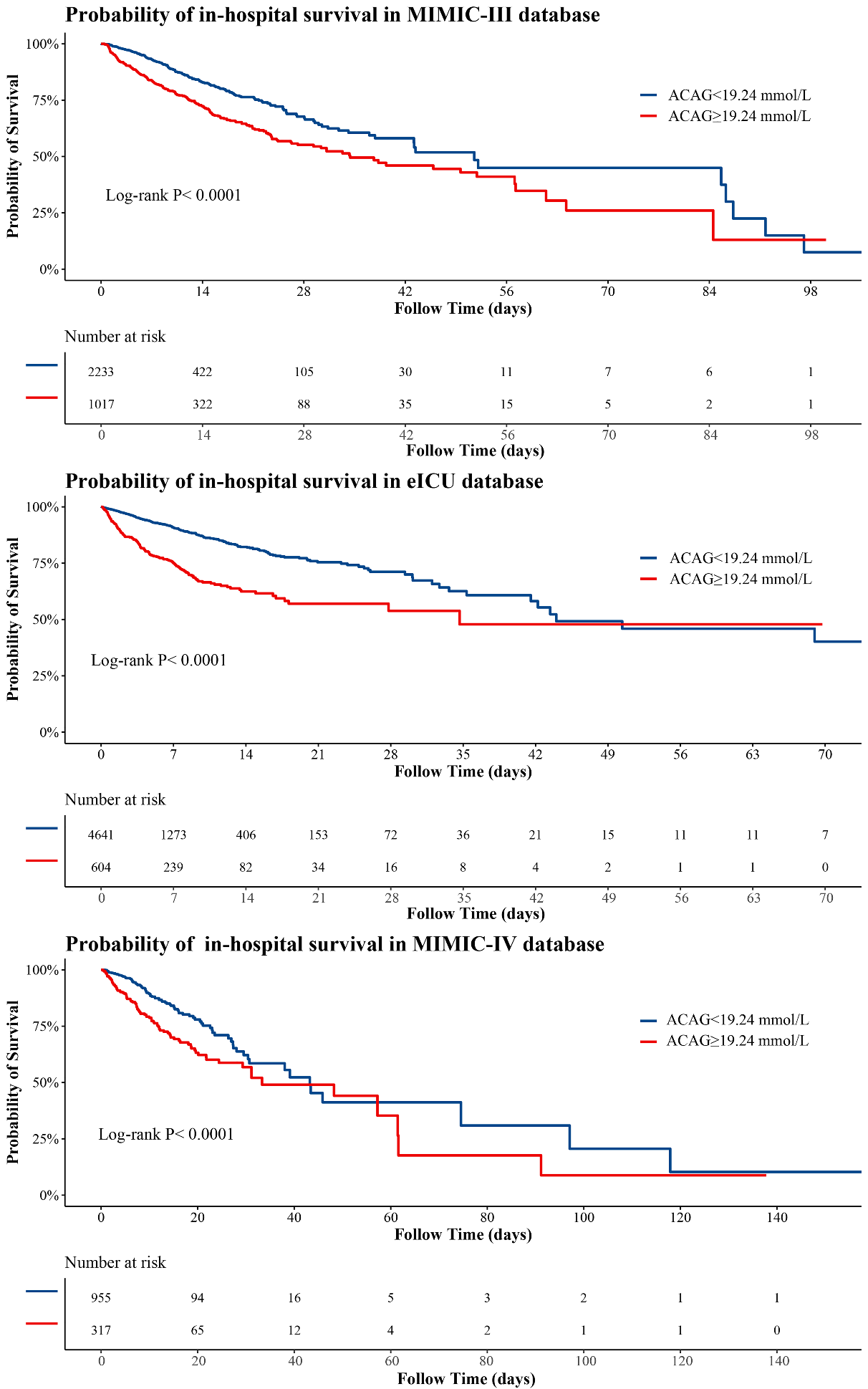
**
